# Effect of common *OPRM1, COMT, SLC6A4, ABCB1*, and *CYP2B6* polymorphisms on perioperative analgesic and propofol demands on patients subjected to thyroidectomy surgery

**DOI:** 10.1007/s43440-023-00455-7

**Published:** 2023-02-07

**Authors:** Ioanna Soultati, Charikleia Ntenti, Georgia Tsaousi, Chryssa Pourzitaki, Dimitris Gkinas, Evanthia Thomaidou, Spiros Alexandrakis, Theodosios Papavramidis, Antonis Goulas

**Affiliations:** 1 Department of Anesthesiology and Intensive Care Unit, School of Medicine, Faculty of Health Sciences, AHEPA University Hospital, Aristotle University of Thessaloniki, Thessaloniki, Greece; 2 1st Laboratory of Pharmacology, School of Medicine, Faculty of Health Sciences, Aristotle University of Thessaloniki, Thessaloniki, Greece; 3 Laboratory of Clinical Pharmacology, School of Medicine, Faculty of Health Sciences, Aristotle University of Thessaloniki, University Campus, 54124 Thessaloniki, Greece; 4 1st Propedeutic Department of Surgery, School of Medicine, Faculty of Health Sciences, AHEPA University Hospital, Aristotle University of Thessaloniki, Thessaloniki, Greece

**Keywords:** Remifentanil, Propofol, OPRM1, COMT, SLC6A4, ABCB1, CYP2B6

## Abstract

**Background:**

Perioperative anesthetic and/or analgesic demand present considerable variation, and part of that variation appears to be genetic in origin. Here we investigate the impact of common polymorphisms in *OPRM1, COMT, SLC6A4, ABCB1,* and *CYP2B6* genes, on the intra-operative consumption of remifentanil and propofol, as well as the postoperative analgesic needs, in patients subjected to thyroidectomy surgery.

**Methods:**

We conducted a prospective cohort study with 90 patients scheduled to undergo elective thyroidectomy, under total intravenous anesthesia achieved by target control infusion (TCI) of propofol and remifentanil. Postoperative analgesics were administered by protocol and on-demand by the individual patient. Genotyping was established by PCR–RFLP methods. Genotyping data, intra-operative hemodynamics, and total consumption of remifentanil and propofol, as well as postoperative analgesic needs and pain perception, were recorded for each individual.

**Results:**

Patients with the *ABCB1* 3435TT genotype appeared to experience significantly less pain within one hour post-operatively, compared to C carriers [mean VAS (SD) = 0.86 (1.22) vs. 2.42 (1.75); *p* = 0.017], a finding limited to those seeking rescue analgesic treatment. Intra-operatively, homozygotes patients for the minor allele of *OPRM1* A118G and *CYP2B6* G516T appeared to consume less remifentanil [mean (SD) = 9.12 (1.01) vs. 13.53 (5.15), for *OPRM1* 118GG and A carriers] and propofol [median (range) = 14.95 (11.53, 1359.5) vs. 121.4 (1.43, 2349.4), for *CYP2B6* 516TT and G carriers, respectively] but the difference was not statistically significant in our sample.

**Conclusions:**

The *ABCB1* C3435T polymorphism appears to affect the postoperative perception of surgical pain among patients with low pain threshold. The small number of minor allele homozygotes for the *OPRM1* A118G and *CYP2B6* G516T polymorphisms precludes a definitive conclusion regarding the inclusion of the latter in a TCI-programming algorithm, based on the results of this study.

**Clinical trial registration number:**

ACTRN12616001598471.

**Supplementary Information:**

The online version contains supplementary material available at 10.1007/s43440-023-00455-7.

## Introduction

Remifentanil is a synthetic μ-opioid receptor (OPRM1) agonist with rapid onset and short duration of action. It displays fast biotransformation by blood esterases and its context-sensitive half-time (time needed to half its blood concentration following the end of administration) is very short, independent of the duration of infusion, and apparently unaffected by liver or kidney function impairment [1]. As an μ-opioid receptor agonist, remifentanil exerts its antinociceptive effect by activating noradrenergic and serotonergic descending inhibitory pathways and by inhibiting the synaptic activation of secondary afferent spinothalamic pathways by Aδ and C fibers at the dorsal horn of the spinal cord [2]. Thus, remifentanil efficacy depends on its ability to cross the blood–brain barrier and could be modulated by P-glycoprotein (ABCB1) activity, as is the case with most opioids [3].

Propofol is an intravenous anesthetic that acts on GABA_A_ receptors as an agonist. It is extensively metabolized, primarily in the liver, but also in the kidneys and the small intestine, mainly by CYP2B6 and UDP glucuronyl transferases (UDPs) [4]. It displays complex pharmacodynamics and shows a synergistic interaction with remifentanil, with respect to both hypnotic and analgesic endpoints [5].

Remifentanil and propofol are usually administered perioperatively by continuous, commonly target-controlled infusion (TCI), in which the infusion rate needed to achieve a predetermined target concentration is calculated by a computing device based on pharmacokinetic (PK) and pharmacodynamic (PD) modeling and patient anthropometric characteristics [6, 7]. More precise, patient-oriented TCI programming could conceivably benefit from the discovery of genetic biomarkers of remifentanil and propofol drug efficacy and optimize perioperative care. In addition,

In contrast to other commonly used opioid drugs which have had their fair share of pharmacogenetic studies [8, 9], data relevant to remifentanil are scarce. The HTTLPR triallelic polymorphism of the *SLC6A4* gene, representing a long/short allele in its promoter region, combined with a MspI restriction site in the extra 43 bp segment of the long allele, was the first polymorphism ever associated with remifentanil efficiency in alleviating experimental thermal pain in healthy volunteers [10], with carriers of the double short allele, or low expressing genotype displaying lower pain perception following bolus intravenous administration of the drug.

The *COMT* Val158Met polymorphism appears in several opioid pharmacogenetic studies either alone or in combination with other polymorphisms, selected as a candidate gene by virtue of its effect on COMT activity in vitro, associated in turn with the availability of μ opioid receptors in certain areas of the brain (basal ganglia, thalamus) [11]. Work on remifentanil includes studies on the pain sensitivity of healthy volunteers [12], on the efficacy of pain relief of preterm newborns after endotracheal intubation [13], and on the intensity of postoperative acute, chronic, or experimental pain following cardiac surgery, together with the OPRM1 gene (*OPRM1*) A118G (rs1799971) polymorphism.

The common rs1045642 polymorphism of the P glycoprotein-encoding gene (*ABCB1* C3435T) was associated with remifentanil consumption and clinical efficacy during elective surgery [14], and with the stabilizing effect of the drug on the hemodynamics of women undergoing cesarian section, as well as on the adaptation of their newborn babies [15]. In addition, *ABCB1* C3435T was recently shown to affect heat-pain threshold in opioid-free adults with chronic pain [16].

*CYP2B6* G516T is a component polymorphism of the common CYP2B6*6 haplotype (as well as others, less common ones) [17]. It was linked to aberrant splicing and low expression of CYP2B6 and was associated with suboptimal biotransformation of propofol, among other drugs [18].

The aim of this study was to examine the possible association of the aforementioned polymorphisms with the intra-operative consumption of remifentanil and propofol through a TCI protocol, and the postoperative analgesic needs and pain perception, in a group of successive patients undergoing total thyroidectomy, using a minimally invasive surgical approach.

## Materials and methods

### Study participants

One hundred and one successively recruited patients (49 from the 3rd Department of Surgery and 40 from the 1st Propedeutic Department of Surgery, AHEPA University Hospital; 12 from the Interbalkan Medical Center, Thessaloniki, Greece) were initially enrolled in this prospective observational study. Participants were all aged 18 years or older, classified according to American Society of Anesthesiology Physical Status (ASA-PS) 1–2, with normal thyroid hormone levels, scheduled to undergo elective thyroidectomy for malignancy, benign disease, or hormonal disease not responsive to medical management. Rules of anonymity and the protection of patients’ personal data were strictly observed throughout the study. Exclusion criteria were age < 18 years, ASA PS ≥ 3, analgesic use up to one week prior to surgery, emergency thyroidectomy, severe thyroid hormone levels disturbance, pregnancy, and diagnosis of a personality disorder. The study protocol was approved by the Scientific Committee of AHEPA University Hospital of Thessaloniki (322/20-5-2016) and the Bioethics Committee of the School of Medicine, Aristotle University of Thessaloniki (316/6-7-2016).

### Study protocol

The evening before the scheduled operation, the anxiety level of each participant was rated according to the Hamilton Anxiety Scale [19]. On the day of surgery, the subjects were premedicated with oral diazepam (5−10 mg). On arrival at the operating theatre, routine monitoring involving electrocardiography, pulse oximetry, and non-invasive blood pressure measurements at 5-min intervals were instituted for each patient and baseline recordings were obtained. Thereafter, a peripheral venous catheter was inserted for intravenous (iv) fluid replacement. Additionally, Bispectral Index (BIS) monitoring (Aspect Medical Systems, Natick, MA) was implemented to guide the anesthesia depth. Anesthesia induction was performed by target-controlled infusion (TCI) of propofol (Schneider pharmacokinetic model) with effect-site concentration set at 4–5 μg/mL, while endotracheal intubation with a proper-sized cuffed endotracheal tube was facilitated following a single dose of cisatracurium (0.2 mg/kg).

Anesthesia was maintained with TCI propofol (effect-site concentration of 3 μg/mL) adjusted by increments of 0.5 mg/mL targeting a BIS value between 40 and 60. Intra-operative analgesia was achieved by TCI remifentanil (1.5–3 μg/mL), adjusted to manage hemodynamic changes exceeding 20% of the baseline values. Normothermia, as assessed by the placement of a mid-esophageal temperature probe, was ensured throughout the surgical procedure.

At the start of skin closure, propofol infusion was discontinued, while as soon as skin suturing was completed remifentanil infusion was also terminated. Before awakening, all patients were given paracetamol 1 g iv and ondansetron 4 mg to ensure postoperative analgesia and protection from postoperative nausea and vomiting, respectively. As soon as clinical signs of an adequate level of consciousness and satisfactory breathing efforts were evidenced, the endotracheal tube was removed, and then all patients were transferred to the post-anesthesia care unit (PACU). After at least a one-hour stay in the PACU, they were discharged to the surgical ward.

The severity of pain at the surgical site was assessed by Visual Analogue Scale (VAS) grading as 0 for the absence of pain and 10 for the worst pain imaginable, during PACU stay (15, 30, and 60 min) and at 2 and 6 h after surgery completion. An anesthesiologist—a member of the research team—ensured that a dedicated protocol for postoperative analgesia management was properly implemented. Rescue analgesics involving paracetamol (1–4 g total, iv) supplemented by parecoxib (40–80 mg total, iv) or lornoxicam (8–16 mg total, iv) were prescribed when clinically notable pain perception (VAS ≥ 4) was encountered upon the predefined time-points of assessment. Tramadol boluses (100 mg total, iv) were administered upon demand to control persistent pain. Intra-operative remifentanil and propofol consumption, as well as total postoperative analgesic requirements being transformed to morphine equivalents (mg), were duly recorded [20–23].

### Genotyping

DNA was extracted from peripheral blood with a commercial DNA extraction kit (Quick-DNA Miniprep Kit, Zymo Research, product number: D3025 Irvine, CA, USA). All polymorphisms were genotyped with previously published PCR–RFLP methods (Table 1) [24–28]. Genotyping was validated with negative and positive controls, by omitting DNA in the reaction mix and by using independently genotyped samples from previous studies, respectively.

**Table 1:** Primers and conditions for PCR–RFLP genotyping

Polymorphism	Primers	Annealing temperature	Restriction enzyme (incubation temperature)	Alleles and corresponding restriction fragments (bp)	Reference
*ABCB1* C3435T (rs1045642)	F: CTCACAGTAACTTGGCAG R: CTTACATTAGGCAGTGAC	52 °C	*Mbo*I (37 °C)	C: 172; 80; 63 T: 235; 80	Gbandi et al. [24]
*COMT* Val158Met (rs4680)	F: TCGTGGACGCCGTGATTCAGG R: AGGTCTGACAACGGGTCAGGC	62 °C	*Hin1*II or *Nla*III (37 oC)	Val: 136; 81 Met: 96; 81; 40	Kunugi et al. [25]
*CYP2B6* G516T (rs3745274)	F: GGTCTGCCCATCTATAAAC R: CTGATTCTTCACATGTCTGCG	56 °C	*Bse*NI or *Bsr*I (65 °C)	G: 268; 241; 17 T: 509; 17	Lang et al. [26]
*OPRM1* A118G (rs1799971)	F: GGTCAACTTGTCCCACTTAGATCGC R: AATCACATACATGACCAGGAAGTTT	60 °C	*BstU*I (60 °C) or *Bsh1236*I (37 °C)	A: 193 G: 169; 24	Zhang et al. [27]
*SLC6A4* HTTLPR (rs4795541)	F: TCCTCCGCTTTGGCGCCTCTTCC R: TGGGGGTTGCAGGGGAGATCCTG	60 °C	NA^a^	L: 512 S: 469	Hooten et al. [28]
*SLC6A4* rs25531	As above	As above	*Msp*I (37 °C)	L_A_: 512 L_G_: 402; 110 S: 469	As above

^a^Not applicable

### Statistical analysis

All continuous variables were tested for normality with the Kolmogorov-Smirnoff test. Deviation of genotype distributions from the Hardy–Weinberg equilibrium was tested with the *χ*^2^ goodness-of-fit test. The effect of each polymorphism on time- and weight-normalized logarithmically transformed remifentanil consumption was tested with ANCOVA, using age, sex, height, Hamilton anxiety scale, and time- and weight-normalized propofol consumption as covariates. The effect of polymorphisms on time- and weight-normalized propofol consumption was examined with the Jonckheere-Terpstra test, due to the wide deviation of propofol consumption from normality which could not be corrected with logarithmic transformation (Figure S1), initially using all three genotypes of each polymorphism as independent variables, and pairwise comparisons as required. The same type of analysis was applied to test the effect of genotypes on VAS, at different time points, from 15 to 360 min following extubation. Spearman’s correlation tests were used to assess the correlation between intra-operative and postoperative drug consumption with VAS at all time points. IBM SPSS statistics 27 was the latest version used for statistical analyses. Bonferroni correction for five polymorphisms and three dependent variables (remifentanil, propofol, and postoperative demands) returns a significance limit of 0.003.

## Results

Patients’ demographic characteristics and genotype distributions for the five polymorphisms are shown in Table 2. For 11 patients, anesthetic/ analgesic drug consumption data were not recorded, and/or DNA was not isolated for various reasons. *OPRM1* A118G and *CYP2B6* G516T genotyping failed for an additional one and 11 patients, respectively. All genotype distributions were consistent with the Hardy–Weinberg equilibrium.

**Table 2:** Patient’s demographic, peprioperative, and genetic characteristics

Variable	Median, range^a^
Age (years)	51.0, 19–78
Sex (female, male)	71, 19
BMI (kg/m^2^)	27.33, 20.28–39.44
Hamilton depression scale	7.0, 0.0–34.0
Duration of anesthesia (min)	85, 30–175
Remifentanil consumption (μg/min)	12.67, 5.55–28.57
Propofol consumption (mg/min)	34.45, 1.43–2349
Postoperative morphine equivalents (mg)	9.25, 5.9–53.6
*OPRM1* A118G, *n* (%)	
AA	58 (64.4)
AG	28 (31.1)
GG	4 (4.4)
Hardy–Weinberg	*p*^b^ = 0.792
*COMT* Val158Met, *n* (%)	
ValVal	20 (22.2)
ValMet	48 (53.3)
MetMet	22 (24.4)
Hardy–Weinberg	*p*^b^ = 0.451
*ABCB1* C3435T, *n* (%)	
CC	22 (24.4)
CT	53 (58.9)
TT	15 (16.7)
Hardy–Weinberg	*p*^b^ = 0.079
*SLC6A4* HTTLPR triallelic, *n* (%)	
L’L’	23 (25.8)
L’S’	39 (43.8)
S’S’	27 (30.3)
Hardy–Weinberg	*p*^b^ = 0.512
*CYP2B6* G516T, *n* (%)	
GG	41 (51.9)
GT	32 (40.5)
TT	6 (7.6)
Hardy–Weinberg	*p*^b^ = 0.997

^a^for continuous variables only

^b^*χ*^2^ of goodness-of-fit for the deviation from Hardy–Weinberg equilibrium

Intra- and postoperative analgesic/anesthetic drug consumption, as well as VAS pain ratings, are summarized in Table 2. When time-normalized for the duration of anesthesia, drug consumption distributions all deviated from normality, with propofol consumption exhibiting the most skewed distribution, as already mentioned (Figure S1). Post-, but not intra-operative analgesic consumption was positively correlated with Hamilton anxiety scale ratings (*p* = 0.001; Table S1), and with VAS ratings at all time points (Table S2). VAS distributions also deviated significantly from normality.

**Fig. 1:**
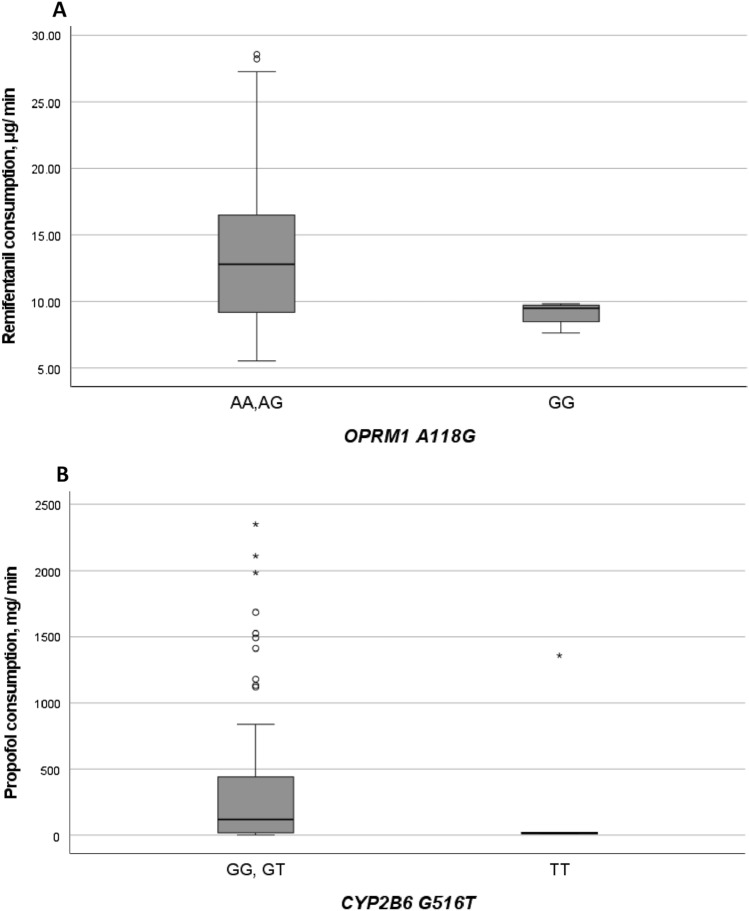
Distributions of intra-operative remifentanil and propofol consumptions, stratified according to the genotypes of the *OPRM1* A118G (**A**) and *CYP2B6* G516T (**B**) polymorphisms, respectively. Shaded boxes: Interquartile range; whiskers: 95% confidence interval; horizontal bars: median values; circles: outliers; asterisks: extreme values, defined as values deviating from the limits of the interquartile range by more than 3 times its magnitude (SPSS)

The effect of the five polymorphisms on intra- and postoperative drug consumption is shown in Table 3. No significant differences were observed between patients carrying different genotypes, for any polymorphism. An apparently lower intra-operative remifentanil consumption observed with patients carrying the *OPRM1* A118GG genotype in comparison to those carrying the AA and AG genotypes (Fig. 1A; S2A) did not reach statistical significance (*p* = 0.082 for GG vs. AA and AG combined; *t* test following logarithmic transformation). Similarly, *CYP2B6* 516TT genotype carriers appeared to consume less propofol compared to *CYP2B6* 516GG and GT carriers intra-operatively (Fig. S2B), but, here again, the difference was not significant (*p* = 0.063 for TT vs. GG and GT combined; Fig. 1B).

**Table 3:** Average drug consumption stratified according to genotype for each polymorphism

Genotype	Remifentanil, μg/min (SD)	Propofol, mg/min (SD)	Postoperative morphine equivalents, mg (SD)
*OPRM1* A118G			
AA	12.99 (4.62)	307.4 (529.6)	15.37 (8.94)
AG	14.59 (6.15)	351.4 (571.6)	14.44 (5.31)
GG	9.12 (1.01)	160.6 (116.0)	12.00 (5.42)
p	0.10^a^	0.42^b^	0.73^b^
*COMT* Val158Met			
ValVal	14.05 (6.22)	276.7 (431.8)	13.28 (5.76)
ValMet	13.77 (5.32)	375.9 (606.5)	14.64 (7.56)
MetMet	11.68 (3.32)	226.8 (429.3)	16.71 (9.79)
p	0.41^a^	0.20^b^	0.15^b^
*ABCB1* C3435T			
CC	12.15 (3.67)	277.9 (439.5)	14.10 (4.84)
CT	13.80 (5.84)	314.5 (594.1)	15.33 (9.21)
TT	13.21 (4.17)	368.2 (422.5)	14.72 (6.14)
p	0.54^a^	0.25^b^	0.96^b^
*SLC6A4* HTTLPR biallelic			
LL	13.42 (5.37)	405.2 (634.9)	13.85 (5.15)
LS	12.43 (4.20)	280.9 (501.1)	15.27 (5.65)
SS	14.93 (6.31)	294.7 (479.2)	15.68 (12.79)
p	0.48^a^	0.99^b^	0.60^b^
*SLC6A4* HTTLPR triallelic			
L’L’	13.54 (5.49)	362.9 (630.3)	13.50 (4.97)
L’S’	12.59 (4.26)	274.5 (451.3)	15.45 (5.83)
S’S’	14.11 (6.11)	342.0 (564.4)	15.57 (11.65)
p	0.66^a^	0.64^b^	0.89^b^
*CYP2B6* G516T			
GG	12.94 (4.48)	413.5 (654.8)	14.49 (7.75)
GT	13.38 (4.82)	305.3 (425. 5)	14.47 (5.99)
TT	9.89 (2.78)	239.2 (548.3)	16.75 (6.52)
p	0.11^a^	0.51^b^	0.52^b^

^a^ANCOVA test, with age, height, sex, Hamilton scale, and time- and weight-normalized propofol consumption as covariates; *p* values refer to the use of log-transformed values

^b^Jonckheere-Terpstra test

**Fig. 2:**
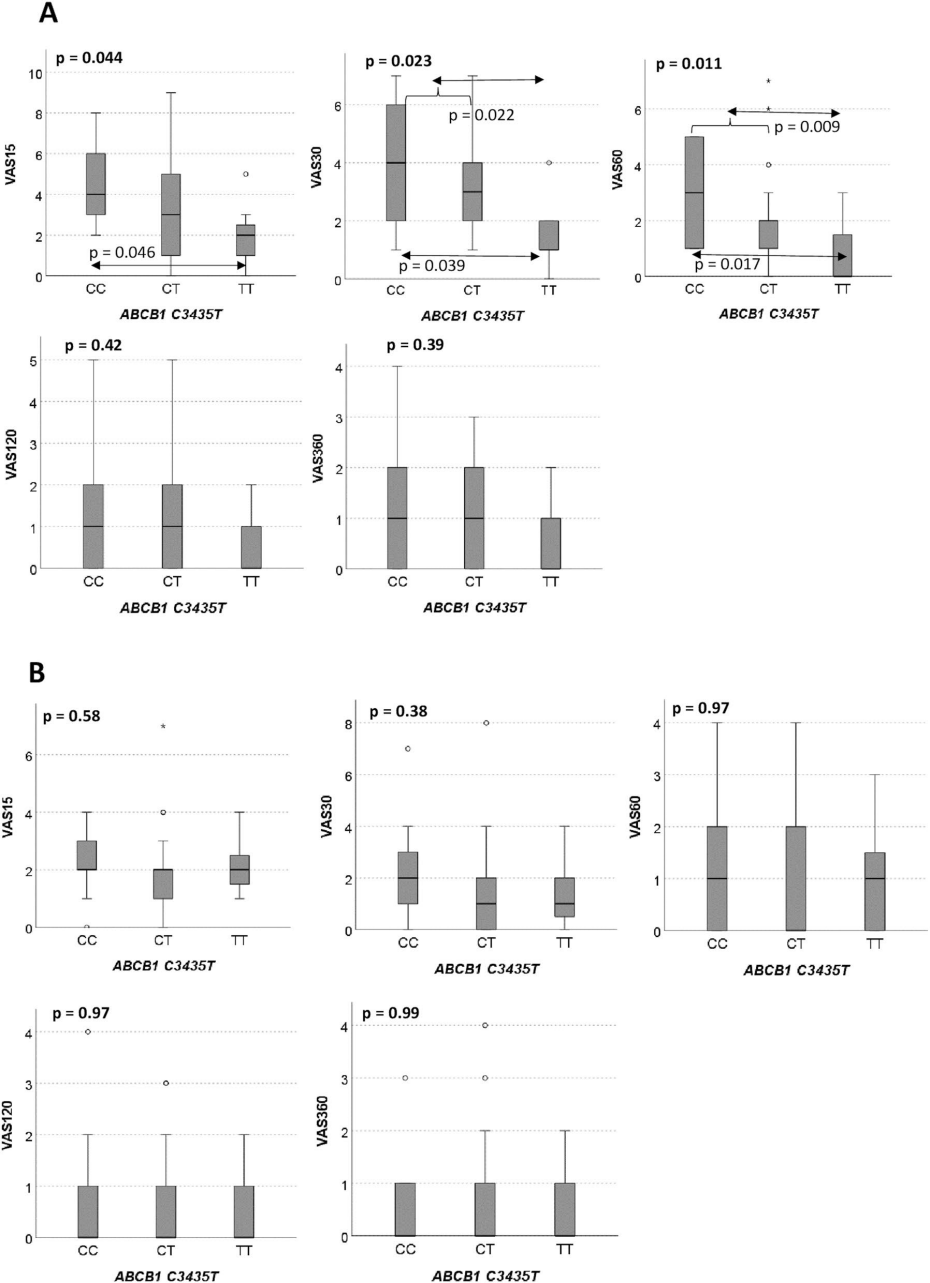
VAS ratings of post-surgical pain, at *t* = 15, 30, 60, 120, and 360 min. **A** patients requiring rescue medication (*n* = 38; CC = 9, CT = 22, TT = 7); **B** patients not requiring rescue medication (*n* = 52; CC = 13, CT = 31, TT = 8). Shaded boxes: Interquartile range; whiskers: 95% confidence interval; horizontal bars: median values; circles: outliers; asterisks: extreme values. VAS distributions for each genotype were compared at each time point with the Jonckheere-Terpstra test. *p* values for three-genotype comparisons are in bold; pairwise comparisons are indicated by two headed arrows (only Bonferroni-adjusted significant *p* values are shown); hooks indicate use of the recessive model for the T allele (TT vs. CC + CT)

The effect of each polymorphism on VAS ratings obtained at 15, 30, 60, 120 and 360 min after completion of surgical procedures was probed next. Results are displayed in Table 4. While no significant associations were recorded overall, the distribution of VAS ratings at time points *t* = 15, 30 and 60 min appeared to be inversely related to the presence of the *ABCB1* 3435 T allele in the patients’ genotype (Fig. S3). As a significant number of patients (*n* = 38; 42.2%) required rescue analgesic treatment suggesting a lower pain threshold (Table S3), the analysis was repeated following stratification accordingly. As shown in Fig. 2, this apparent effect observed with the entire sample (Fig. S3) reached statistical significance for those patients requiring additional analgesia, but not for those who did not *ABCB1.* C3435T genotype distributions did not differ between the two subgroups (Table S4). Finally, because anxiety is known to affect the perception of pain and Hamilton rating scale values were positively correlated with postoperative analgetic consumption, we have examined the effect of the *ABCB1* C3435T genotype on Hamilton ratings, with negative results (Table S5).

**Table 4:** VAS ratings (SD) stratified according to genotype for each polymorphism

Genotype	VAS15_min_	VAS_30min_	VAS_60min_	VAS_120min_	VAS_360min_
*OPRM1* A118G					
AA (58)	2.59 (2.10)	2.22 (2.04)	1.41 (1.56)	0.91 (1.34)	0.79 (1.16)
AG (28)	2.50 (2.12)	2.25 (1.92)	1.64 (1.75)	0.89 (1.07)	0.79 (0.96)
GG (4)	2.75 (1.50)	2.25 (1.26)	1.25 (1.26)	0.25 (0.50)	0.25 (0.50)
p^a^	0.72	0.57	0.55	0.99	0.83
*COMT* Val158Met					
ValVal (20)	2.26 (1.85)	2.21 (1.78)	1.58 (1.54)	0.79 (0.98)	0.63 (0.90)
ValMet (48)	2.65 (1.95)	2.25 (2.08)	1.44 (1.62)	0.87 (1.33)	0.74 (1.19)
MetMet (22)	2.77 (2.47)	2.32 (1.92)	1.55 (1.65)	0.95 (1.29)	0.86 (1.08)
p^a^	0.38	0.70	0.94	0.85	0.45
*ABCB1* C3435T					
CC (22)	3.18 (2.06)	2.82 (2.17)	1.86 (1.78)	0.95 (1.46)	0.82 (1.18)
CT (53)	2.45 (2.20)	2.21 (1.98)	1.47 (1.61)	0.94 (1.23)	0.81 (1.12)
TT (15)	2.07 (1.34)	1.47 (1.25)	0.93 (1.10)	0.53 (0.83)	0.53 (0.74)
p^a^	0.09	0.06	0.13	0.63	0.62
*SLC6A4* HTTLPR biallelic					
LL	2.46 (2.45)	1.88 (1.96)	1.38 (2.00)	0.96 (1.46)	0.78 (1.09)
LS	2.65 (2.02)	2.37 (2.05)	1.56 (1.53)	0.77 (1.17)	0.70 (1.04)
SS	2.64 (1.71)	2.45 (1.79)	1.50 (1.26)	1.05 (1.13)	0.86 (1.21)
p^a^	0.33	0.20	0.32	0.54	0.75
*SLC6A4* HTTLPR triallelic					
L’L’(23)	2.30 (2.31)	1.87 (2.10)	1.22 (1.93)	0.91 (1.44)	0.77 (1.11)
L’S’(39)	2.87 (2.12)	2.46 (2.00)	1.72 (1.62)	0.79 (1.24)	0.69 (1.06)
S’S’(27)	2.44 (1.74)	2.30 (1.79)	1.41 (1.22)	1.00 (1.07)	0.85 (1.13)
p^a^	0.33	0.21	0.25	0.35	0.50
*CYP2B6* G516T					
GG (41)	2.68 (1.96)	2.30 (1.98)	1.46 (1.47)	0.78 (1.17)	0.73 (1.04)
GT (32)	2.77 (1.99)	2.26 (1.93)	1.52 (1.50)	0.94 (1.18)	0.77 (1.09)
TT (6)	3.67 (3.39)	3.33 (2.81)	2.50 (2.88)	1.83 (2.23)	1.33 (1.75)
p^a^	0.72	0.77	0.75	0.35	0.64

^a^Jonckheere-Terpstra test

## Discussion

While the primary objective of this study was to examine the effect of common polymorphisms previously related to opioid and propofol efficiency on intra-operative remifentanil and propofol requirements (consumption) of by patients who had undertaken elective thyroidectomy, its main—and rather unexpected—finding was a time-dependent effect of *ABCB1* C3435T on post-surgical pain perception; patients carrying the TT genotype apparently reported significantly lower pain perception compared to C carriers, at times 15, 30 and 60 min following the end of the surgical procedure, when VAS ratings are highest and most varied. Furthermore, that effect was detected only among those patients requiring rescue analgesic medication, i.e., with a low pain threshold. We argue that the fact that the significance of this association appears to be time-dependent and rather consistent throughout most of the postoperative period argues against it being a chance finding. Since neither VAS ratings nor the *ABCB1* C3435T polymorphism is associated with intra-operative consumption of remifentanil or propofol, one should look into postoperative analgesic consumption or the mechanism of pain perception for an explanation. As VAS ratings are consistently higher in patients requiring additional (rescue) postoperative treatment (Table S5), the former must reflect either differences in the interindividual efficiency of standard postoperative paracetamol or in some physiologic parameter causally related to pain perception. Paracetamol is known to induce ABCB1 expression [29] but was not shown to function as its substrate. On the other hand, a recently published report by Hooten et al. [16] suggested that the *ABCB1* 3435TT genotype is associated with increased threshold to heat pain in opioid-free adults with chronic pain. The authors attributed that apparent effect on alterations in the efflux of endogenous opioid peptides from the brain, previously shown to be mediated by ABCB1 [30]. Even though more needs to be done to corroborate those findings and articulate a more detailed working hypothesis as to a probable underlying mechanism, it is encouraging that in our study the *ABCB1* 3435TT genotype is also associated with a lower perception of pain and that this effect is made apparent only among those patients predisposed to higher pain sensitivity (i.e., in need of rescue medication). That would be consistent with a situation where the concentration of opioids in the CNS becomes limiting and is thus affected by transport systems in the blood–brain barrier, including ABCB1.

Our findings with respect to the effect of the five gene polymorphisms on the intra-operative remifentanil and propofol consumption were less promising, even though it is perhaps worth mentioning that all four carriers of the *OPRM1* 118GG genotype consumed low amounts of remifentanil, and so did five out of six patients with the *CYP2B6* 516TT genotype with respect to propofol. A small number of studies have examined the association of the same *OPRM1* polymorphism with intra-operative opioid (fentanyl, sulfentanil, or morphine) requirements, all of them involving obstetrics analgesia [reviewed in [31], and in [32], with inconclusive results, even though carriage of the G allele appeared to somewhat increase opioid efficacy, which is not contradictory to our findings. According to our literature search, no study has examined the effect of *OPRM1* A118G on remifentanil requirements thus far, with the exception of a single report highlighting a protective effect of the maternal G allele on neonates whose mothers had received remifentanil in the context of labor anesthesia [15]. Despite propofol being an established substrate for CYP2B6, the effect of *CYP2B6* G516T on propofol pharmacokinetics remains controversial, with some evidence supporting the association of the T allele with decreased metabolism and, consequently, lower dose requirements of propofol [33, 34], which is also somewhat consistent to our present findings. The limited number of TT genotypes in this study precludes any further analysis, however, especially since other factors, such as gender and age, are known to also affect CYP2B6 activity [17]. On the other hand, we found no apparent effect of either the *OPRM1* or the *CYP2B6* polymorphism on postoperative analgesic consumption, which can be explained by the very short context-sensitive half-time of remifentanil on one hand, and the very limited use of opioids and/or CYP2B6 substrates post-operatively.

According to previous studies of the postoperative use of morphine [35, 36] *COMT* 158MetMet carrier patients should display a lower opioid requirement compared to *COMT* 158Val carriers. Overall, however, published findings on the effects of *COMT* Val158Met on opioid (fentanyl, hydromorphone, morphine, butorphanole, or meperidine) requirements or pain threshold, alone or in combination with *OPRM1* A118G [13, 37, 38], are characterized by inconsistencies, which our findings are unable to resolve since no significant association was observed with respect to analgesic consumption in our study.

The HTTLPR polymorphism has been examined in association with opioid use in the past, by virtue of 5-HT involvement in the modulation of pain signaling at the spinal level, but no significant differences in daily opioid consumption were found among patients suffering from chronic pain, carrying different genotypes [28]. With respect to non-opioid analgesics, the HTTLPR polymorphism was associated with poorer response to carbamazepine in idiopathic trigeminal neuralgia patients [39], but not with triptan response in patients with cluster headaches [40]. In our study, the HTTLPR polymorphism was not associated with any perioperative related outcome whatsoever, be that remifentanil consumption, propofol consumption, pain perception as assessed by VAS, or postoperative consumption of analgesics in morphine equivalents.

Our rather small sample size and the limited number of *OPRM1* A118G and *CYP2B6* G516T minor allele homozygotes are obvious limitations of our study, and so could be the over-representation of women, since propofol metabolism is known to be affected by sex, among other things [41]. On the contrary, the single type of elective surgery, with the same standardized surgical and anesthesiologic procedures, and the ethnic homogeneity of the participating patient population have presumably reduced other confounding effects.

In conclusion, we have provided evidence suggestive of an effect of the *ABCB1* C3435T polymorphism on the postoperative perception of surgical pain which, combined with recent findings concerning heat pain perception in opioid-free patients with chronic pain, points to a significant contribution of ABCB1 efflux pump to the process of central pain management by the organism. Our data concerning the association of *OPRM1* A118G and *CYP2B6* G516T with intra-operative remifentanil and propofol consumption does not support their inclusion in a TCI-programming algorithm at this point.

## Supplementary Information

Below is the link to the electronic supplementary material.

Supplementary file1 (DOCX 123 KB)

## Data Availability

The authors have also submitted supplemental data and declare that if needed they can provide all the data concerning the present manuscript.

## Abbreviations

5-HT: 5-Hydroxytryptamine
ABCB1: ATP binding cassette subfamily B member 1
ANCOVA: Analysis of covariance
ANZCTR: Australian New Zealand clinical trials registry
ASA-PS: American society of anesthesiology physical status
BIS: Bispectral index
COMT: Catechol-O-methyltransferase
CYP: Cytochrome P450
DNA: Deoxyribonucleic acid
GABA: Gamma-aminobutyric acid
HTTLPR: Serotonin-transporter-linked promoter region
OPRM1: μ-Opioid receptor 1
PACU: Post anesthesia care unit
PCR-RFLP: Polymerase chain reaction-restriction fragment length polymorphism
SD: Standard deviation
SLC6A4: Solute carrier family 6 member 4
TCI: Targeted control infusion
UDP glycuronyl transferases: Uridine 5'-diphospho-glucuronosyltransferase
VAS: Visual analogue scale

## Declarations

### Permission to reproduce material from other sources

The authors declare that there is no reproduced material from other sources in the present manuscript.

### Clinical trial registration

The trial was submitted with the acronym “PERRETT”(PErioperative Remifentanyl REquiremenT Thyroidectomy) for inclusion in the Australian New Zealand Clinical Trials Registry (ANZCTR) and has been registered and allocated the ACTRN: ACTRN12616001598471.
