# Supplementary material for: Effect of common OPRM1, COMT, SLC6A4, ABCB1, and CYP2B6 polymorphisms on perioperative analgesic and propofol demands on patients subjected to thyroidectomy surgery
Source: Pharmacol Rep. 2023 Feb 7;75(2):386–96. doi: 10.1007/s43440-023-00455-7 (PMC10060341; doi:10.1007/s43440-023-00455-7)
Supplement: Supplementary file 1 — Supplementary file1 (DOCX 123 KB) [file 43440_2023_455_MOESM1_ESM.docx]

SUPPLEMENT

| **Table S1** Correlation of drug consumption with the Hamilton anxiety scale ratings of patients | | |
| --- | --- | --- |
| Drug | Spearman’s ρ | p |
| Remifentanil^a^ | -0.022 | 0.840 |
| Propofol^a^ | 0.080 | 0.455 |
| Morphine equivalents^b^ | 0.339 | 0.001 |

^a^ time- and weight-normalized

^b^ total

| **Table S2** Correlation of post-operative drug administered, in morphine equivalents, with VAS ratings of the patients, at different time points | | |
| --- | --- | --- |
|  | Spearman’s ρ | p |
| VAS_15min_ | 0.332 | 0.001 |
| VAS_30min_ | 0.452 | <0.001 |
| VAS_60min_ | 0.459 | <0.001 |
| VAS_120min_ | 0.370 | <0.001 |
| VAS_360min_ | 0.344 | <0.001 |

| **Table S3** Comparison of VAS ratings (SD) between patients who received paracetamol only and those who required rescue analgesics | | | |
| --- | --- | --- | --- |
|  | Paracetamol only  n = 52 | Paracetamol +^a^  n = 38 | p^b^ |
| VAS_15min_ | 1.92 (1.30) | 3.45 (2.55) | < 0.001 |
| VAS_30min_ | 1.63 (1.70) | 3.05 (2.01) | < 0.001 |
| VAS_60min_ | 1.00 (1.28) | 2.13 (1.16) | < 0.001 |
| VAS_120min_ | 0.61 (1.00) | 1.24 (1.42) | 0.023 |
| VAS_360min_ | 0.57 (0.94) | 1.03 (1.20) | 0.047 |

^a^ additional (rescue) administration of parecoxib (n = 27), lornoxicam (n = 10), tramadol (n = 5) alone, or in combination

^b^ Mann-Whitney test

| **Table S4** Comparison of *ABCB1* C3435T distributions between patients who received paracetamol only and those who required rescue analgesics; n (%) | | |
| --- | --- | --- |
| *ABCB1*C3435T genotype | Paracetamol only, n (%) | Paracetamol +^a^, n (%) |
| CC | 13(25.0) | 9 (23.7) |
| CT | 31 (59.6) | 22 (57.9) |
| TT | 8 (15.4) | 7 (18.4) |
| Total | 52 (100.0) | 38 (100.0) |
| p^b^ | 0.929 |  |

^a^ additional (rescue) administration of parecoxib (n = 27), lornoxicam (n = 10), tramadol (n = 5) alone, or in combinationABCB1

^b^ *χ*^2^ test of independence

| **Table S5.** Effect of *ABCB1* C3435T polymorphism on Hamilton anxiety scale | |
| --- | --- |
| *ABCB1* C3435T genotype | Hamilton rating scale |
| CC | 10.14 (7.54) |
| CT | 9.49 (7.46) |
| TT | 10.64 (7.85) |
| p^a^ | 0.922 |

^a^ Jonckheere-Tepstra test

A

B
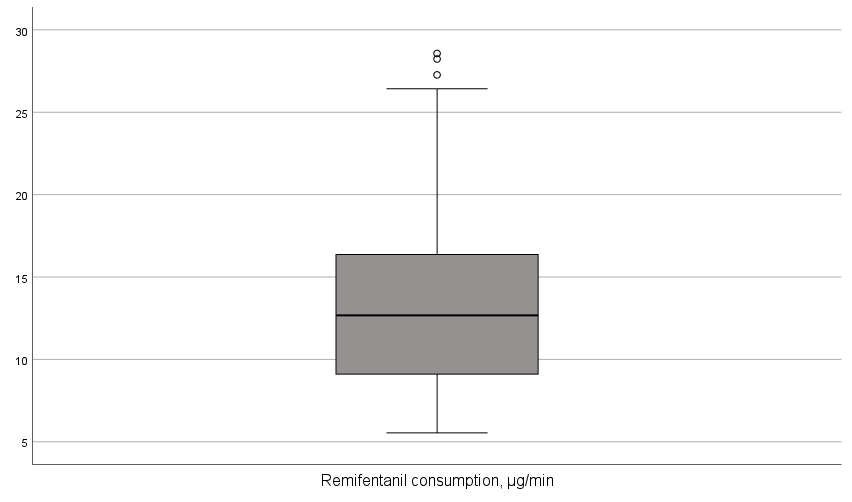


C
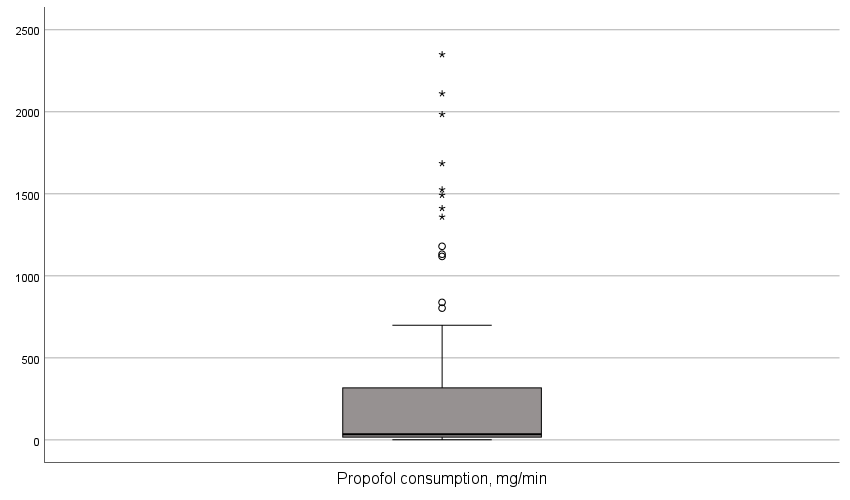


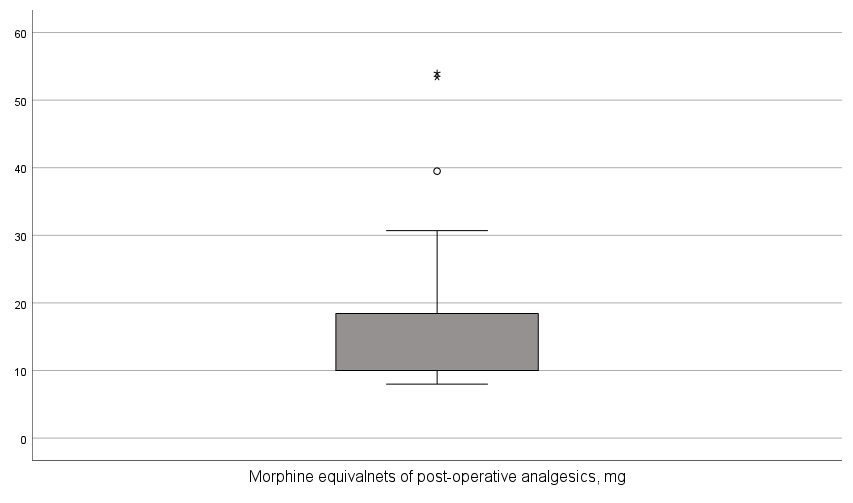


**Figure S1.** Distributions of intra-operative remifentanil (A), propofol (B), and post-operative analgesic drug (C) consumptions


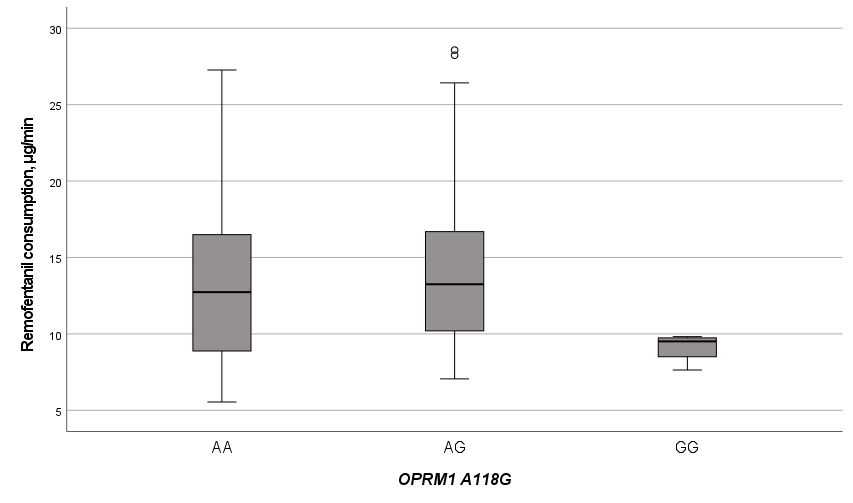


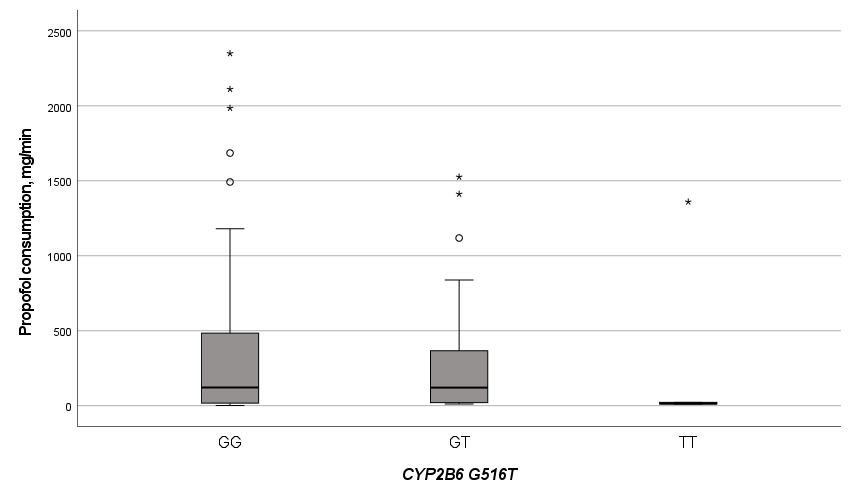


B

**Figure S2**. Distributions of intraoperative remifentanil and propofol consumptions, stratified according to the genotypes of the *OPRM1* A118G (A) and *CYP2B6* G516T (B) polymorphisms, respectively.


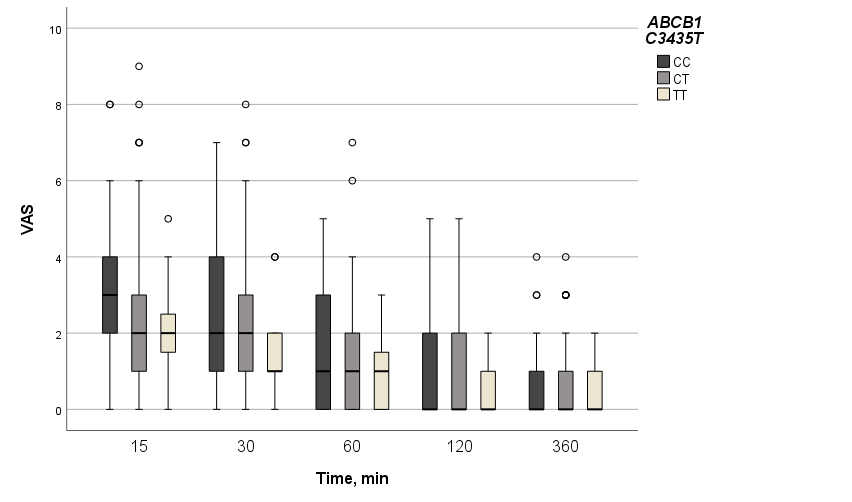


**Figure S3**. Effect of the *ABCB1* C3435T polymorphism on the VAS distributions of the patients at different time points following the conclusion of the operation.

All boxplots: Shaded boxes: interquartile range; horizontal bands: median values; whiskers: 95% confidence intervals; circles: outliers; asterisks: extreme values
